# Neurological and systemic adverse event reporting patterns for lecanemab: A global WHO VigiAccess analysis

**DOI:** 10.1097/MD.0000000000051045

**Published:** 2026-11-06

**Authors:** Nasser M. Alorfi, Alanood S. Algarni

**Affiliations:** a Pharmacology and Toxicology Department, College of Pharmacy, Umm Al-Qura University, Makkah, Saudi Arabia.

**Keywords:** adverse drug reactions, Alzheimer disease, amyloid-related imaging abnormalities, lecanemab, monoclonal antibody, pharmacovigilance, VigiAccess

## Abstract

Lecanemab is an anti-amyloid monoclonal antibody approved for early Alzheimer disease. Evaluation of spontaneous reports may complement clinical-trial evidence by identifying real-world adverse-event reporting patterns. This study aimed to characterize lecanemab-associated adverse drug reaction reports in the World Health Organization VigiAccess database at the System Organ Class (SOC) and Preferred term (PT) levels. A retrospective descriptive pharmacovigilance analysis was conducted using VigiAccess data from database inception through 13 September 2026. Frequencies and percentages were calculated for SOCs, PTs, demographic characteristics, geographic regions, and reporting years. Wilson 95% confidence intervals were calculated for principal reporting proportions. VigiAccess contained 3212 individual safety reports comprising 5358 adverse drug reaction events. Nervous system disorders constituted the largest SOC (n = 1827; 34.10%; 95% confidence interval: 32.84–35.38), followed by general disorders and administration-site conditions (n = 1015; 18.94%; 95% confidence interval: 17.92–20.01). The most frequently reported PTs were headache (n = 512; 9.56%), amyloid-related imaging abnormality–edema/effusion (n = 360; 6.72%), amyloid-related imaging abnormality–microhemorrhages and hemosiderin deposits (n = 326; 6.08%), chills (n = 281; 5.24%), pyrexia (n = 272; 5.08%), and infusion-related reaction (n = 235; 4.39%). Females accounted for 52.21% of reports, patients aged ≥ 65 years for 62.95%, and the Americas for 80.23%. Most reports (95.73%) were recorded during 2024 to 2026. Neurological, amyloid-related imaging abnormality, systemic, and infusion-associated events were prominent in spontaneous reporting for lecanemab. These findings describe reporting patterns and should not be interpreted as incidence rates or causal associations. Continued patient-level and exposure-adjusted surveillance is required to further characterize lecanemab safety.

## 1. Introduction

Alzheimer’s disease (AD) is a chronic, progressive neurodegenerative disorder that primarily involves widespread damage to the cerebral cortex and hippocampus, resulting in gradual deterioration of memory and cognition.^[1,2]^ It remains a major global health challenge, characterized by continuous neurodegeneration and deterioration in cognitive functions, particularly memory, reasoning, and behavior.^[3,4]^

Pathologically, AD is marked by the accumulation of extracellular neuritic plaques and intracellular neurofibrillary tangles, accompanied by the degeneration of basal forebrain cholinergic neurons.^[5,6]^ The plaques consist of amyloid-*β* peptides derived from the amyloid precursor protein, while the tangles are formed by hyperphosphorylated tau, a microtubule-associated protein.^[7,8]^ Under normal conditions, amyloid-*β* is efficiently cleared from the brain, but failure of this clearance leads to its accumulation and subsequent neurotoxicity.^[9,10]^ Similarly, disruption in the balance of tau phosphorylation results in its detachment from microtubules and aggregation within neurons.^[11,12]^ The coexistence of amyloid-β accumulation and hyperphosphorylated tau formation constitutes the core neuropathological features driving the progression of AD.^[13,14]^

Lecanemab is a Food and Drug Administration -approved humanized Immunoglobulin G1monoclonal antibody that selectively targets and binds with high affinity to soluble amyloid-β protofibrils, the neurotoxic aggregates implicated in the early stages of AD.^[15,16]^ It was developed as part of a new generation of anti-amyloid immunotherapies aimed at disease modification rather than symptomatic control. Clinical trials have demonstrated that lecanemab can remove amyloid-*β* plaques, slow cognitive decline, and maintain a low incidence of amyloid-related imaging abnormalities in early AD.^[17,18]^ As real-world use expands, post-marketing surveillance is critical to identify additional or unexpected adverse effects beyond those observed under controlled trial conditions.

The World Health Organization Programme for International Drug Monitoring collects individual case safety reports submitted through national pharmacovigilance systems worldwide in VigiBase, which is maintained by the Uppsala Monitoring Centre. VigiAccess is the publicly accessible interface of VigiBase and provides aggregated information according to active ingredient, Medical Dictionary for Regulatory Activities System Organ Class and Preferred Term, patient characteristics, geographic region, and reporting year. VigiAccess was selected because its multinational coverage permits characterization of global lecanemab reporting patterns. In contrast, the United States Food and Drug Administration Adverse Event Reporting System and EudraVigilance primarily represent reports submitted within the United States and European regulatory systems, respectively. Nevertheless, VigiAccess provides aggregated rather than case-level data and cannot independently establish causality, incidence, comparative risk, or confirmed safety signals.

Given the recent introduction and expanding clinical use of lecanemab, updated evaluation of spontaneous pharmacovigilance reports may provide clinically relevant information regarding its real-world reporting profile. Therefore, this study aimed to characterize lecanemab-associated reporting patterns in World Health Organization (WHO) VigiAccess through analysis of adverse drug reaction (ADR) events at the System Organ Class and Preferred Term levels and evaluation of patient sex, age group, geographic distribution, and temporal reporting trends. Particular attention was given to neurological, amyloid-related imaging abnormality, cerebrovascular, and infusion-related events.

## 2. Methodology

### 2.1. Study design

A retrospective, descriptive pharmacovigilance study was conducted to characterize spontaneously reported adverse reactions associated with lecanemab. The analysis included all lecanemab reports available through the WHO VigiAccess database from database inception through 13 September 2026. The study was designed to describe reporting patterns at the System Organ Class (SOC) and preferred term (PT) levels rather than estimate adverse-event incidence, establish causality, or quantify comparative risk.

### 2.2. Data source and rationale for database selection

Data were obtained from VigiAccess, the publicly accessible interface of VigiBase, the WHO global database of individual case safety reports maintained by the Uppsala Monitoring Centre on behalf of the WHO Programme for International Drug Monitoring. VigiBase receives reports submitted through national pharmacovigilance systems in multiple countries and regions.

VigiAccess was selected because its multinational coverage permitted examination of the global reporting profile of lecanemab. This coverage differs from region-specific pharmacovigilance systems such as the United States, Food and Drug Administration Adverse Event Reporting System and EudraVigilance. However, VigiAccess provides aggregated rather than case-level information and does not provide access to complete report narratives, individual clinical histories, reporter assessments, exposure denominators, or patient-level covariates.

### 2.3. Search strategy and data extraction

VigiAccess was searched using the active ingredient term “lecanemab.” The final extraction was performed on 13 September 2026. All reports associated with lecanemab that were available in the database on the extraction date were included. No restrictions were applied according to patient age, sex, geographic region, reporting year, seriousness, or reporter type.

The extracted variables comprised the total number of individual safety reports, distribution of reported ADR events by Medical Dictionary for Regulatory Activities (MedDRA) SOC, frequencies of individual MedDRA PTs, patient sex, patient age group, geographic region, and annual number of reports. SOC categories and PTs were transcribed as displayed in VigiAccess on the extraction date.

VigiAccess did not disclose the specific MedDRA release number applied to the publicly displayed dataset. Consequently, SOC and PT classifications were reported according to the MedDRA terminology implemented in VigiAccess on 13 September 2026. No terms were independently recoded or reassigned by the investigators. The absence of a publicly specified MedDRA version was recognized as a limitation affecting exact reproducibility.

### 2.4. Definitions and study outcomes

An individual safety report was defined as a report associated with lecanemab in VigiAccess. A reported ADR event was defined as an individual MedDRA-coded reaction contained within a safety report. Because 1 report could contain more than 1 ADR event, the number of reported events could exceed the number of individual safety reports.

The primary outcome was the distribution of reported ADR events across MedDRA SOCs. Secondary outcomes included the most frequently reported PTs, with particular attention to neurological, cerebrovascular, amyloid-related imaging abnormality, and infusion-related events. Additional outcomes included distributions according to patient sex, age group, geographic region, and reporting year.

### 2.5. Statistical analysis

Categorical variables were summarized using frequencies and percentages. SOC and PT level percentages were calculated by dividing the number of events within each SOC or PT by the total number of reported ADR events and multiplying by 100. Demographic, geographic, and yearly percentages were calculated using the total number of individual safety reports as the denominator. Unknown or unreported demographic categories were retained in their respective denominators to avoid overestimating the proportions of reports with known characteristics.

For SOC and selected PT proportions, 95% confidence intervals were calculated using the Wilson score method. These intervals were used to describe the precision of the observed reporting proportions and were not interpreted as estimates of population incidence or treatment related risk. Individual ADR events were not necessarily statistically independent because multiple events could originate from the same safety report.

Reporting odds ratios, proportional reporting ratios, and other disproportionality measures were not calculated because the publicly accessible VigiAccess output did not provide the complete drug event and comparator event contingency data required for valid disproportionality analysis. In addition, direct comparisons with aducanumab or donanemab would be influenced by differences in approval dates, cumulative exposure, geographic availability, prescribing patterns, monitoring requirements, and duration of postmarketing surveillance. Accordingly, the analysis was restricted to descriptive reporting patterns and did not classify reported events as confirmed pharmacovigilance signals or infer comparative safety.

### 2.6. Ethical considerations

Ethical approval and informed consent were not required because the study used publicly accessible, aggregated, and anonymized secondary pharmacovigilance data. No identifiable or case-level patient information was accessed or analyzed.

## 3. Results

### 3.1. Overall reporting

As of 13 September 2026, VigiAccess contained 3212 individual safety reports associated with lecanemab. Collectively, these reports included 5358 ADR events classified across 26 MedDRA System Organ Classes (SOCs). The number of ADR events exceeded the number of individual reports because a single safety report could contain more than 1 reported reaction. An average of 1.67 ADR events was recorded per report.

### 3.2. Distribution by system organ class

Nervous system disorders constituted the largest SOC, accounting for 1827 of the 5358 ADR events (34.10%; 95% confidence interval (CI): 32.84–35.38). This was followed by general disorders and administration-site conditions (n = 1015; 18.94%; 95% CI: 17.92–20.01), injury, poisoning and procedural complications (n = 419; 7.82%; 95% CI: 7.13–8.57), psychiatric disorders (n = 326; 6.08%; 95% CI: 5.48–6.76), and gastrointestinal disorders (n = 312; 5.82%; 95% CI: 5.23–6.48).

Investigations accounted for 217 events (4.05%), followed by skin and subcutaneous-tissue disorders (n = 205; 3.83%), musculoskeletal and connective tissue disorders (n = 193; 3.60%), infections and infestations (n = 152; 2.84%), and vascular disorders (n = 149; 2.78%). The complete distribution of reported ADR events across the 26 SOCs is presented in Table 1.

**Table 1:** Distribution of lecanemab-associated ADR events by MedDRA system organ class.

MedDRA system organ class	ADR events, n	Percentage	95% CI
Nervous system disorders	1827	34.10%	32.84–35.38
General disorders and administration-site conditions	1015	18.94%	17.92–20.01
Injury, poisoning and procedural complications	419	7.82%	7.13–8.57
Psychiatric disorders	326	6.08%	5.48–6.76
Gastrointestinal disorders	312	5.82%	5.23–6.48
Investigations	217	4.05%	3.55–4.61
Skin and subcutaneous-tissue disorders	205	3.83%	3.34–4.37
Musculoskeletal and connective-tissue disorders	193	3.60%	3.14–4.14
Infections and infestations	152	2.84%	2.42–3.32
Vascular disorders	149	2.78%	2.37–3.26
Respiratory, thoracic and mediastinal disorders	103	1.92%	1.59–2.33
Cardiac disorders	95	1.77%	1.45–2.16
Eye disorders	89	1.66%	1.35–2.04
Metabolism and nutrition disorders	78	1.46%	1.17–1.81
Renal and urinary disorders	37	0.69%	0.50–0.95
Ear and labyrinth disorders	28	0.52%	0.36–0.75
Neoplasms benign, malignant and unspecified, including cysts and polyps	26	0.49%	0.33–0.71
Immune system disorders	25	0.47%	0.32–0.69
Blood and lymphatic-system disorders	14	0.26%	0.16–0.44
Surgical and medical procedures	14	0.26%	0.16–0.44
Product issues	10	0.19%	0.10–0.34
Hepatobiliary disorders	8	0.15%	0.08–0.29
Reproductive-system and breast disorders	8	0.15%	0.08–0.29
Social circumstances	5	0.09%	0.04–0.22
Congenital, familial and genetic disorders	2	0.04%	0.01–0.14
Endocrine disorders	1	0.02%	0.00–0.11
Total	5358	100.00%	–

### 3.3. Preferred term-level distribution

Preferred Term-level analysis demonstrated that headache was the most frequently reported event (n = 512; 9.56%; 95% CI: 8.80–10.37). Amyloid-related imaging abnormality–edema/effusion was the second most frequently reported PT (n = 360; 6.72%; 95% CI: 6.08–7.42), followed by amyloid-related imaging abnormality–microhemorrhages and hemosiderin deposits (n = 326; 6.08%; 95% CI: 5.48–6.76).

Frequently reported systemic and infusion-associated PTs included chills (n = 281; 5.24%), pyrexia (n = 272; 5.08%), infusion-related reaction (n = 235; 4.39%), and fatigue (n = 216; 4.03%). Other frequently reported neurological or neuropsychiatric events included dizziness (n = 176; 3.28%), confusional state (n = 160; 2.99%), fall (n = 96; 1.79%), tremor (n = 93; 1.74%), and unspecified amyloid-related imaging abnormalities (n = 85; 1.59%).

Clinically important cerebrovascular and neurological PTs included cerebral hemorrhage (n = 67; 1.25%), cerebrovascular accident (n = 44; 0.82%), seizure (n = 39; 0.73%), brain edema (n = 32; 0.60%), and cerebral microhemorrhage (n = 29; 0.54%). The most frequently reported and selected clinically important PTs are presented in Table 2.

**Table 2:** Most frequently reported and selected clinically important MedDRA preferred terms associated with lecanemab.

MedDRA preferred term	ADR events, n	Percentage	95% CI
Headache	512	9.56%	8.80–10.37
Amyloid-related imaging abnormality–edema/effusion	360	6.72%	6.08–7.42
Amyloid-related imaging abnormality–microhemorrhages and hemosiderin deposits	326	6.08%	5.48–6.76
Chills	281	5.24%	4.68–5.87
Pyrexia	272	5.08%	4.52–5.70
Infusion-related reaction	235	4.39%	3.87–4.97
Fatigue	216	4.03%	3.54–4.59
Dizziness	176	3.28%	2.84–3.80
Confusional state	160	2.99%	2.56–3.48
Nausea	156	2.91%	2.49–3.40
Fall	96	1.79%	1.47–2.18
Tremor	93	1.74%	1.42–2.12
Vomiting	90	1.68%	1.37–2.06
Asthenia	90	1.68%	1.37–2.06
Amyloid-related imaging abnormalities, unspecified	85	1.59%	1.28–1.96
Influenza-like illness	81	1.51%	1.22–1.87
Rash	76	1.42%	1.13–1.77
Cerebral hemorrhage	67	1.25%	0.99–1.58
Diarrhea	61	1.14%	0.89–1.46
Alzheimer-type dementia	61	1.14%	0.89–1.46
Cerebrovascular accident	44	0.82%	0.61–1.10
Seizure	39	0.73%	0.53–0.99
Brain edema	32	0.60%	0.42–0.84
Cerebral microhemorrhage	29	0.54%	0.38–0.78

### 3.4. Demographic distribution

Females accounted for 1677 reports (52.21%), whereas males accounted for 1160 reports (36.11%). Sex was unknown or unreported in 375 reports (11.67%).

Reports most frequently involved patients aged ≥ 75 years (n = 1115; 34.71%), followed by patients aged 65–74 years (n = 907; 28.24%) and those aged 45–64 years (n = 326; 10.15%). Only 4 reports (0.12%) involved individuals aged 18 to 44 years. Age was unknown or unreported in 860 reports (26.77%). Overall, 2022 reports (62.95%) involved patients aged ≥ 65 years. Complete demographic findings are presented in Table 3.

**Table 3:** Demographic characteristics of individual lecanemab safety reports.

Characteristic	Reports, n	Percentage
Sex		
Female	1677	52.21%
Male	1160	36.11%
Unknown or unreported	375	11.67%
Age group		
18–44 yrs	4	0.12%
45–64 yrs	326	10.15%
65–74 yrs	907	28.24%
≥75 yrs	1115	34.71%
Unknown or unreported	860	26.77%

### 3.5. Geographic distribution

The Americas accounted for most individual safety reports (n = 2577; 80.23%), followed by Asia (n = 462; 14.38%) and Europe (n = 166; 5.17%). Oceania contributed 7 reports (0.22%). The geographic distribution is shown in Table 4.

**Table 4:** Geographic distribution of individual lecanemab safety reports.

Geographic region	Reports, n	Percentage
Americas	2577	80.23%
Asia	462	14.38%
Europe	166	5.17%
Oceania	7	0.22%
Total	3212	100.00%

### 3.6. Temporal distribution

Reporting increased markedly following 2023. One report was recorded in 2021 (0.03%), 5 in 2022 (0.16%), and 131 in 2023 (4.08%). The annual number increased to 979 reports in 2024 (30.48%) and reached 1102 reports in 2025 (34.31%). An additional 994 reports (30.95%) were recorded from 1 January through 13 September 2026. Thus, 3075 of the 3212 reports (95.73%) were recorded during 2024 to 2026. The annual distribution is presented in Table 5.

**Table 5:** Annual distribution of individual lecanemab safety reports.

Reporting yr	Reports, n	Percentage
2021	1	0.03%
2022	5	0.16%
2023	131	4.08%
2024	979	30.48%
2025	1102	34.31%
2026*	994	30.95%
Total	3212	100.00%

ADR = adverse drug reaction.

* Data for 2026 were available only through September 13, 2026, and therefore represent an incomplete reporting year. Percentages were calculated using the total number of individual safety reports (N = 3212) as the denominator. Annual counts represent the number of reports submitted to the database and not the number of ADR events.

## 4. Discussion

This descriptive pharmacovigilance analysis characterized 3212 individual safety reports containing 5358 ADR events associated with lecanemab in VigiAccess through 13 September 2026. Nervous system disorders constituted the largest SOC, accounting for approximately one-third of reported events, followed by general disorders and administration-site conditions. PT-level analysis showed that headache, amyloid-related imaging abnormality–edema/effusion, and amyloid-related imaging abnormality–microhemorrhages and hemosiderin deposits were among the most frequently reported events. These findings provide clinically relevant detail beyond broad SOC classifications and demonstrate that neurological, imaging-related, and infusion-associated reactions represent prominent components of spontaneous reporting for lecanemab.

Headache was the most frequently reported PT, accounting for 512 events. Headache is clinically relevant during lecanemab treatment because it may occur as a nonspecific adverse reaction, as part of an infusion-related reaction, or in association with amyloid-related imaging abnormalities. However, the available aggregated data did not permit determination of the clinical context, timing, severity, imaging findings, or relationship between headache and amyloid-related imaging abnormality (ARIA) within individual reports. Therefore, headache reports should not be assumed to represent ARIA in the absence of corresponding clinical and radiological information.

ARIA-related PTs constituted a substantial component of neurological reporting. Amyloid-related imaging abnormality edema/effusion accounted for 360 events, whereas amyloid-related imaging abnormality microhemorrhages and hemosiderin deposits accounted for 326 events. An additional 85 events were classified as unspecified amyloid-related imaging abnormalities. Brain edema, cerebral microhemorrhage, cerebral hemorrhage, cerebrovascular accident, and seizure were also reported, although less frequently. These findings are pharmacologically plausible because lecanemab binds soluble amyloid-β protofibrils and promotes amyloid removal. Mobilization of amyloid from cerebral tissue and vascular deposits may temporarily alter vascular permeability and integrity, contributing to vasogenic edema or effusion classified as amyloid-related imaging abnormality–edema/effusion and microhemorrhage, hemosiderin deposition, or superficial siderosis classified as ARIA-H.

ARIA is a recognized safety concern across the anti-amyloid monoclonal-antibody class, including lecanemab, aducanumab, and donanemab. Nevertheless, the present findings should not be interpreted as demonstrating that lecanemab carries a higher or lower ARIA risk than these agents. Valid comparative assessment would require exposure-adjusted data, consistent follow-up periods, comparable imaging protocols, and patient-level information regarding amyloid burden, apolipoprotein E genotype, anticoagulant use, baseline microhemorrhages, and treatment duration. Differences in regulatory approval dates, geographic availability, prescribing volume, and monitoring practices also preclude direct comparison of raw spontaneous-report counts among these agents.

The observed ARIA-related reporting pattern supports the clinical importance of established risk-mitigation measures during lecanemab treatment. magnetic resonance imaging assessment before treatment and at recommended intervals during therapy facilitates the detection of asymptomatic or symptomatic ARIA. Clinical vigilance is especially relevant among apolipoprotein E 4 carriers and patients with preexisting cerebral microhemorrhages or other indicators of cerebral amyloid angiopathy. Particular caution is also warranted when evaluating patients receiving anticoagulant or antithrombotic therapy because intracranial hemorrhagic events may have serious clinical consequences. However, VigiAccess does not provide the case-level information needed to determine whether the reported hemorrhagic events occurred in genetically susceptible patients or those receiving concomitant antithrombotic treatment.

General disorders and administration-site conditions constituted the second-largest SOC. At the PT level, chills, pyrexia, infusion-related reaction, fatigue, asthenia, and influenza-like illness were frequently reported. This pattern is compatible with the intravenous administration of a therapeutic monoclonal antibody and with infusion-related reactions recognized during lecanemab treatment. These reactions may reflect transient immune activation and can occur during or shortly after infusion. Nevertheless, the aggregated database did not provide sufficient information regarding infusion number, onset, severity, management, recurrence, or treatment discontinuation.

Gastrointestinal events represented 5.82% of all reported ADR events, with nausea, vomiting, and diarrhea among the most frequently recorded PTs. Psychiatric disorders accounted for 6.08% of events, while confusional state was among the leading individual PTs. Interpretation of these findings requires caution because nausea and vomiting may occur during systemic or infusion-related reactions, while confusion and other neuropsychiatric symptoms may reflect an adverse reaction, progression of the underlying neurodegenerative disease, an intercurrent illness, or other concomitant treatment. The absence of detailed clinical narratives prevents differentiation among these explanations.

Females accounted for 52.21% of individual reports, compared with 36.11% for males, while sex was unknown in 11.67%. Reports concerning patients aged ≥ 65 years represented 62.95% of all reports, although age was unknown in 26.77%. These distributions are broadly compatible with the population treated for early Alzheimer disease, but they should not be interpreted as evidence that female or older patients experience a higher treatment-related risk. Sex- and age-specific exposure denominators were unavailable, and spontaneous reporting may be influenced by disease prevalence, treatment utilization, clinical monitoring, and reporting behavior.

The geographic distribution was highly concentrated in the Americas, which accounted for 80.23% of reports, followed by Asia, Europe, and Oceania. This concentration is more likely to reflect differences in regulatory timing, market availability, cumulative treatment exposure, pharmacovigilance participation, and reporting practices than differences in biological susceptibility. Because VigiAccess did not provide country-level exposure denominators, the geographic findings cannot be used to compare regional incidence or safety.

Reporting increased markedly after 2023, with 95.73% of reports recorded during 2024 to 2026. This temporal increase coincided with the expanding regulatory approval, clinical availability, and surveillance of lecanemab. The increased number of reports may consequently reflect greater cumulative exposure and heightened clinical awareness rather than a change in the intrinsic safety of the medicine. Furthermore, the 2026 data covered only the period through 13 September and cannot be directly compared with complete calendar years.

The PT-level findings improve the clinical utility of the analysis by identifying the specific reactions underlying the broad SOC distributions. However, the results represent reporting proportions rather than incidence rates, causal effects, or measures of comparative risk. The predominance of neurological, ARIA-related, and infusion-associated events is consistent with the pharmacological action and established adverse-effect profile of lecanemab, but these reporting patterns should be interpreted as hypothesis-generating observations requiring confirmation through patient-level pharmacoepidemiological studies, prospective registries, and comparative analyses with appropriate exposure denominators.

## 5. Conclusion

Analysis of WHO VigiAccess data showed that neurological, amyloid-related imaging abnormality, systemic, and infusion-associated events constituted prominent components of spontaneous reporting for lecanemab. Headache, ARIAedema/effusion, ARIA–microhemorrhages and hemosiderin deposits, chills, pyrexia, and infusion-related reactions were among the most frequently reported Preferred Terms. These findings are consistent with the pharmacological action and recognized adverse-effect profile of lecanemab but represent reporting patterns rather than incidence rates or confirmed causal associations. Continued pharmacovigilance, supported by patient-level studies, exposure-adjusted analyses, and appropriate comparisons with other anti-amyloid monoclonal antibodies, is needed to further characterize the real-world safety of lecanemab.

## 6. Limitations

This study has limitations inherent to spontaneous pharmacovigilance data, including underreporting, stimulated or selective reporting, duplicate submissions, incomplete clinical information, and differences in reporting practices across countries and periods. Because the number of patients exposed to lecanemab was unavailable, the reported proportions cannot be interpreted as incidence rates or measures of treatment-related risk.

VigiAccess provides aggregated rather than case-level data. Therefore, causality could not be assessed, and information regarding dose, treatment duration, time to onset, clinical outcomes, concomitant medicines, apolipoprotein E genotype, anticoagulant use, and imaging findings was unavailable. In addition, the 5358 ADR events originated from 3212 reports and were not necessarily statistically independent; consequently, the Wilson confidence intervals describe reporting proportions only.

Reporting odds ratio and proportional reporting ratio analyses could not be performed because VigiAccess did not provide the complete comparator-event data required for valid disproportionality analysis. Direct comparison with aducanumab or donanemab was also limited by differences in exposure, approval dates, availability, and monitoring practices. Furthermore, VigiAccess did not disclose the specific MedDRA version used, and the 2026 data covered only the period through 13 September. Accordingly, the findings should be interpreted as descriptive and hypothesis-generating rather than evidence of causal or comparative safety associations.

## Author contributions

**Software:** Nasser M. Alorfi.

**Supervision:** Nasser M. Alorfi.

**Validation:** Nasser M. Alorfi.

**Visualization:** Nasser M. Alorfi.

**Writing – original draft:** Nasser M. Alorfi.

**Writing – review & editing:** Nasser M. Alorfi, Alanood S. Algarni.

**Resources:** Alanood S. Algarni.

## References

[1] Deture MA Dickson DW. The neuropathological diagnosis of Alzheimer’s disease. Mol Neurodegener. 2019;14:32–18. 10.1186/s13024-019-0333-5 PMC6679484 31375134

[2] Masters CL Bateman R Blennow K Rowe CC Sperling RA Cummings JL. Alzheimer’s disease. Nat Rev Dis Primers. 2015;1:15056–18. 10.1038/nrdp.2015.56 27188934

[3] Oh K Yoon JS Suk H. Learn-explain-reinforce: counterfactual reasoning and its guidance to reinforce an alzheimer’s disease diagnosis model. IEEE Trans Pattern Anal Mach Intell. 2023;45:4843–57. 10.1109/TPAMI.2022.3197845 35947563

[4] Hazelton JL Fittipaldi S Fraile-Vazquez M. Thinking versus feeling: how interoception and cognition influence emotion recognition in behavioural-variant frontotemporal dementia, Alzheimer’s disease, and Parkinson’s disease. Cortex. 2023;163:66–79. 10.1016/j.cortex.2023.02.009 PMC11177281 37075507

[5] Nisbet RM Polanco JC Ittner LM Götz J. Tau aggregation and its interplay with amyloid-β. Acta Neuropathol. 2015;129:207–20. 10.1007/s00401-014-1371-2 PMC4305093 25492702

[6] Huang HC Jiang ZF. Accumulated amyloid-β peptide and hyperphosphorylated tau protein: relationship and links in alzheimer’s disease. JAD. 2009;16:15–27. 10.3233/JAD-2009-0960 19158417

[7] Bloom GS. Amyloid-β and tau: the trigger and bullet in Alzheimer disease pathogenesis. JAMA Neurol. 2014;71:505–8. 10.1001/jamaneurol.2013.5847 PMC12908160 24493463

[8] Spires-Jones TL Hyman BT. The intersection of amyloid beta and tau at synapses in Alzheimer’s disease. Neuron. 2014;82:756–71. 10.1016/j.neuron.2014.05.004 PMC4135182 24853936

[9] Ondrejcak T Klyubin I Hu NW Barry AE Cullen WK Rowan MJ. Alzheimer’s disease amyloid β-protein and synaptic function. Neuromol Med. 2009;12:13–26. 10.1007/s12017-009-8091-0 19757208

[10] Xin SH Tan L Cao X Yu JT Tan L. Clearance of Amyloid Beta and Tau in Alzheimer’s Disease: from Mechanisms to Therapy. Neurotox Res. 2018;34:733–48. 10.1007/s12640-018-9895-1 29626319

[11] Wegmann S Biernat J Mandelkow E. A current view on Tau protein phosphorylation in Alzheimer’s disease. Curr Opin Neurobiol. 2021;69:131–8. 10.1016/j.conb.2021.03.003 33892381

[12] Mi K Johnson GVW. The role of tau phosphorylation in the pathogenesis of Alzheimer’s disease. Curr Alzheimer Res. 2006;3:449–63. 10.2174/156720506779025279 17168644

[13] Ittner LM Götz J. Amyloid-β and tau — a toxic pas de deux in Alzheimer’s disease. Nat Rev Neurosci. 2010;12:67–72. 10.1038/nrn2967 21193853

[14] Busche MA Hyman BT. Synergy between amyloid-β and tau in Alzheimer’s disease. Nat Neurosci. 2020;23:1183–93. 10.1038/s41593-020-0687-6 PMC11831977 32778792

[15] Honig LS Sabbagh MN van Dyck CH. Updated safety results from phase 3 lecanemab study in early Alzheimer’s disease. Alzheimers Res Ther. 2024;16:105–12. 10.1186/s13195-024-01441-8 PMC11084061 38730496

[16] Chowdhury S Chowdhury NS. Novel anti-amyloid-beta (Aβ) monoclonal antibody lecanemab for Alzheimer’s disease: a systematic review. Int J Immunopathol Pharmacol. 2023;37:3946320231209839. 10.1177/03946320231209839 PMC10617290 37902139

[17] van Dyck CH Swanson CJ Aisen P. Lecanemab in Early Alzheimer’s disease. N Engl J Med. 2023;388:9–21. 10.1056/NEJMoa2212948 36449413

[18] Swanson CJ Zhang Y Dhadda S. A randomized, double-blind, phase 2b proof-of-concept clinical trial in early Alzheimer’s disease with lecanemab, an anti-Aβ protofibril antibody. Alzheimers Res Ther. 2021;13:80–14. 10.1186/s13195-021-00813-8 PMC8053280 33865446

